# Grünholzfraktur des Unterarmschafts – Überbrechen obligatorisch oder fakultativ?

**DOI:** 10.1007/s00113-024-01477-3

**Published:** 2024-09-16

**Authors:** Thomas Petnehazy, Martin Münnich, Ferdinand Füsi, Saskia Hankel, Anna Erker, Elena Friehs, Hesham Elsayed, Holger Till, Georg Singer

**Affiliations:** Universitätsklinik für Kinder- und Jugendchirurgie, Medizinische Universität Graz, Auenbruggerplatz 34, 8036 Graz, Österreich

**Keywords:** Diaphysäre Unterarmfraktur, Gegenkortikalis, Kinder, Jugendliche, Refraktur, Diaphyseal forearm fracture, Substantia compacta, Children, Adolescents, Refracture

## Abstract

**Hintergrund:**

„To break or not to break“ – Wie gehen wir mit der Gegenkortikalis um? Das ist die Frage, die sich die Kindertraumatologie seit vielen Jahren hinsichtlich der Therapie von Grünholzfrakturen des Unterarmschafts stellt.

**Fragestellung:**

Häufigkeit der Grünholzfrakturen des diaphysären Unterarms im Kindes- und Jugendalter; Einfluss des Durchbrechens der Gegenkortikalis auf die Refrakturrate.

**Material und Methode:**

Analyse und Diskussion relevanter Arbeiten, Analyse der Refrakturrate von kindlichen Grünholzfrakturen des Unterarmschafts im eigenen Patientenkollektiv.

**Ergebnisse:**

Grünholzfrakturen treten im Bereich des Unterarmschafts häufig auf, und eine inkomplette Konsolidierung führt zu einer erhöhten Refrakturrate. Im Patientenkollektiv der Autoren von 420 Kindern mit Grünholzfrakturen des Unterarmschafts zeigte sich eine Refrakturrate von 9,5 %, wobei die Rate bei nichtkomplettierten Frakturen signifikant höher war, im Vergleich zur Gruppe der komplettierten Frakturen (15,2 % vs. 3 %). Während in der Subgruppe der konservativ behandelten Grünholzfrakturen (*n* = 234) das Durchbrechen der Gegenkortikalis die Refrakturrate signifikant verringerte, führte das Durchbrechen im Rahmen einer operativen Versorgung mithilfe der elastisch-stabilen intramedullären Nagelung (ESIN) zu keiner Veränderung der Refrakturrate.

**Schlussfolgerungen:**

Im Rahmen der konservativen Therapie von Grünholzfrakturen des diaphysären Unterarms kann das Durchbrechen der Gegenkortikalis empfohlen werden, um die Refrakturrate zu minimieren. Das Komplettieren der Grünholzfraktur scheint im Rahmen einer operativen Behandlung mithilfe der ESIN nicht nötig zu sein.

## Abstract (en)

**Background:**

To break or not to break—That is the question that has been asked in pediatric traumatology for many years regarding the treatment of greenstick fractures of the diaphyseal forearm shaft.

**Objective:**

The frequency of greenstick fractures of the forearm shaft in children and adolescents; the influence of breaking the fracture on the refracture rate.

**Methods:**

Analysis and discussion of relevant articles, analysis of the refracture rate of pediatric greenstick fractures of the forearm shaft in our own patient population.

**Results:**

Greenstick fractures frequently occur in the area of the forearm shaft and incomplete consolidation leads to an increased refracture rate. In the patient collective of the authors of 420 children with greenstick fractures of the diaphyseal forearm, there was a refracture rate of 9.5%; however, the rate for non-completed fractures was significantly higher compared to the group with completed fractures (15.2% vs. 3%). While in the subgroup of conservatively treated fractures (*n* = 234), breaking the intact cortex significantly reduced the refracture rate, breaking the intact cortical bone during surgical treatment with elastic stable intramedullary nailing (ESIN) did not change the refracture rate.

**Conclusion:**

As part of the conservative treatment of greenstick fractures of the diaphyseal forearm, completing the fracture can be recommended in order to lower the refracture rate. Completing the fracture does not appear to be necessary during surgical treatment using ESIN.

„To break or not to break“ – das ist die Frage, die sich die Kindertraumatologie seit vielen Jahren hinsichtlich der Therapie von Grünholzfrakturen des Unterarmschafts stellt. Kaum eine andere Fraktur wird bezüglich ihres therapeutischen Ansatzes so kontrovers diskutiert und unterliegt einer ähnlich dynamischen Entwicklung ihrer Behandlung wie die diaphysäre Grünholzfraktur des kindlichen Unterarmschafts, gilt es doch, bekannte Komplikationen wie Refrakturen und Bewegungseinschränkungen aufgrund persistierender Fehlstellungen zu vermeiden.

Die durch zahlreiche Studien belegte Bandbreite der konservativen Therapieformen von Grünholzfrakturen des Unterarms reicht vom Vertrauen in das wachsende Skelett und dessen Korrekturpotenzial über die geschlossene Reposition und Gipsruhigstellung bis hin zum Durchbrechen der Gegenkortikalis und zur Überführung einer primär per se stabilen Fraktur in eine instabile Fraktur mit allen daraus resultierenden Vor- und Nachteilen [1–4]. Darüber hinaus ist im Laufe der Jahre ein deutlicher Trend zur chirurgischen Sanierung der Unterarmschaftfrakturen, insbesondere zur operativen Versorgung mithilfe der elastisch-stabilen intramedullären Nagelung (ESIN), festzustellen; wohl auch als Zeichen der Limitationen der konservativen Therapie mit der Sorge über persistierende Deformitäten und dem Bedürfnis nach achsengerechter Versorgung [5]. Die veränderten Freizeitgewohnheiten, vermehrte Ausübung von Rasanzsportarten mit höherem Verletzungsrisiko sowie die Ansprüche der Patient*innen und der Angehörigen nach rascher Mobilisierung und gutem funktionellem Ergebnis werden als weitere Gründe für die Zunahme der operativen Therapie angesehen [6].

## Epidemiologie: Wie häufig ist die diaphysäre Unterarmfraktur?

So, wie die Therapieformen der Unterarmfraktur über die Zeit einem Wandel unterliegen, verändert sich auch die Inzidenz der Unterarmfrakturen selbst. Mehrere Studien berichten von einer Steigerung der Inzidenz von über 30 % über die Jahrzehnte hinweg [7, 8]. Körner et al. haben 2019 die Häufigkeit von Frakturen der oberen Extremität bei Kindern und Jugendlichen, die in deutschen Krankenhäusern versorgt wurden, über einem Zeitraum von 16 Jahren untersucht und konnten eine signifikante Erhöhung der Inzidenz von Unterarmschaftfrakturen in der Altersgruppe von 0 bis 14 Jahren mit einem standardisierten Inzidenzverhältnis ≥ 1,44 feststellen [9]. Der Altersgipfel der diaphysären Unterarmfrakturen fand sich in dieser Studie bei 8 Jahren und scheint im Vergleich zu früheren Berichten konstant zu bleiben [10].

Die Inzidenz der Unterarmfrakturen ist im Laufe der letzten Jahrzehnte über 30 % angestiegen

Unterarmfrakturen treten bei Schulkindern (65 %) und Jugendlichen (63 %) deutlich häufiger auf als bei Kleinkindern (42 %) und im Vorschulalter (50 %) [11]. Jungen sind gut doppelt so häufig betroffen wie Mädchen mit in zunehmendem Alter steigender Tendenz [9, 12]. In der aktuellen Literatur variieren die Angaben zum Anteil der Unterarmfrakturen an der Gesamtzahl aller kindlichen Frakturen von 17,8 % bis zu 41 % [13–15]. Die diaphysären Unterarmfrakturen werden mit einer Inzidenz von 3–5,4 % angegeben [15, 16]. Die mit über 82 % häufigste Frakturform des kindlichen Unterarms ist die Grünholzfraktur [17]. Auch bei den diaphysären Unterarmfrakturen beträgt ihr Anteil noch gut 33 % [18].

## Korrekturpotenzial: Was remodelliert der kindliche Unterarmschaft spontan?

Die Behandlung der diaphysären Unterarmschaftfraktur war über Jahrzehnte eine Domäne der konservativen Therapie [4, 5, 19]. Innerhalb altersabhängiger Fehlstellungsgrenzen werden gute Ergebnisse mithilfe der Gipsruhigstellung ohne Reposition beschrieben [2, 20]. Achsabweichungen von bereits 10° können je nach Richtung und Lokalisation zu signifikanten Einschränkungen der Umwendbeweglichkeit führen. Schmittenbecher hat 2005 in seiner Übersichtsarbeit über die Behandlung der Unterarmschaftfrakturen die Limitationen der konservativen Therapie und die Vorteile der operativen Versorgung mithilfe der ESIN bei bestimmten Frakturtypen aufgezeigt [21].

Je jünger die Betroffenen und je distaler die Unterarmfraktur, desto größer ist das Korrekturpotenzial

Bezüglich der Spontankorrektur gilt die goldene Regel: Je jünger die Patient*innen und je distaler die Unterarmfraktur lokalisiert ist, desto größer ist das Korrekturpotenzial [10]. Im Bereich der Diaphyse und des proximalen Unterarms ist dieses jedoch kaum noch vorhanden. Ellenbogennahe Frakturen tolerieren nahezu keine persistierenden Fehlstellungen mehr. Ausnahme von dieser Regel ist das proximale Radiusende, das Achsabweichungen in der Frontal- und Sagittalebene im weiteren Wachstum ausgleichen kann [10].

Kadaverstudien haben gezeigt, dass Angulationen des Unterarmschafts ab 10° zu messbaren Bewegungseinschränkungen führen und ab 20° mit deutlichen funktionellen Defiziten zu rechnen ist [22, 23]. Diese Erkenntnis wurde von Sarmiento et al. 1992 anhand klinischer Untersuchungen validiert [24] und in weiterer Folge auch für die pädiatrische Population bestätigt [25–27]. Nebst kosmetischer Defizite stellt eine persistierende Deformität im diaphysären Bereich des Unterarms v. a. für die Umwendbewegungen ein erhebliches Problem dar [27]. Pro- und Supination sind keine reine Rotationsbewegungen um einen Drehpunkt, sondern vielmehr komplexe Abfolgen von Rotations- und Translationsbewegungen [28]. Jede Deformität, die den interossären Raum im Bereich der Überkreuzungsstelle der beiden Unterarmknochen einengt, führt unweigerlich zu einem Funktionsverlust. Die komplexe Bewegung des Unterarms in Kombination mit seinem anspruchsvollen anatomischen Aufbau macht es schwierig, die genaue Ursache für die Bewegungseinschränkungen zu lokalisieren und diese in weiterer Folge zu korrigieren [26]. Auf die Spontankorrektur von Rotationsfehlstellungen gibt es kaum Hinweise, und die Studienlage ist sehr inkonsistent [29].

Wie Valone et al. 2020 in ihrer Studie zeigen konnten, kommen Kinder mit einem Bewegungsumfang von 35°Extension und 147° Flexion im Ellenbogengelenk sowie 56° Supination und 64° Pronation im Routinealltag, einschließlich Handytelefonieren, Computermaus- und Tastaturbedienung, sehr gut zurecht [30]. Um diese Funktionalität der Umwendbewegungen zu gewährleisten, sollten proximal der Schaftmitte keine Achsfehlstellungen akzeptiert werden. In einer prospektiven Kohortenstudie untersuchten Barvelink et al. 2020 das Korrekturpotenzial von kindlichen Unterarmfrakturen und berichteten über eine durchschnittliche Spontankorrektur von 8° innerhalb eines Jahres bei Kindern mit einem Durchschnittsalter von 9 Jahren [2]. In diese Studie wurden sowohl isolierte Radiusfrakturen als auch Unterarmschaftfrakturen inkludiert; die Frakturlokalisation wurde jedoch nicht berücksichtigt. Das Korrekturpotenzial des diaphysären Unterarmschafts beträgt bis zum Abschluss des Wachstums hingegen „nur“ 1–2° pro Jahr [31–33]. Dies sollte bei der Planung einer konservativen Therapie keinesfalls außer Acht gelassen werden.

Generell haben sich die Empfehlungen von Noonan und Price hinsichtlich der Toleranzgrenzen von Achsabweichungen bei der Behandlung von Unterarmschaftfrakturen bewährt und seit 1998 kaum verändert [34]. Scotcher et al. haben 2022 in ihrem systematischen Review aus initial 2058 Arbeiten als Ergebnis die Toleranzgrenzen in der Sagittalebene um Toleranzgrenzen in der Frontalebene ergänzt [29]. Die Autoren empfehlen tolerable Achsabweichungen für Kinder jünger als 10 Jahre < 15° in der Sagittal- und < 10° in der Frontalebene sowie eine < 50 %ige Translationsfehlstellung. Für Kinder älter als 10 Jahre werden als Toleranzgrenzen < 10° in der Sagittal- und in der Frontalebene sowie ebenfalls eine < 50 %ige Translationsfehlstellung empfohlen [29].

## Was ist bei der Therapie zu beachten?

Soll eine Grünholzfraktur des Unterarmschafts aufgrund ihrer Achsfehlstellung reponiert werden, ist Folgendes zu bedenken und entsprechend vorzugehen:

Nach der Reposition sperrt die in Abheilung befindliche Kortikalis die Heilung der Gegenkortikalis

Die Grünholzfrakturen sind typische Frakturen des wachsenden Skeletts. Sie gehören zu den Biegungsbrüchen und weisen somit immer eine Achsabweichung auf. Die Biegungsbrüche werden in 3 Formen unterteilt: die frühkindliche gestauchte Form (< 5 Jahre), die gebogene Form bekannt als „bowing fracture“ und die klassische Grünholzfraktur, die das zentrale Thema dieser Arbeit darstellt. Bei dieser Frakturform ist die Kortikalis auf der konvexen Seite der Angulation durchgebrochen, aber auf der konkaven Seite lediglich nur angebrochen. Wird die Fraktur reponiert oder innerhalb der weiter oben beschriebenen Toleranzgrenzen belassen, heilt die Fraktur auf der konkaven Seite der Fehlstellung rasch ab, während auf der konvexen Seite die nötige Kompression zur Konsolidierung der Fragmente fehlt. Die in Abheilung befindliche Kortikalis sperrt die Heilung der Gegenkortikalis. Hier kommt es zu einer verzögerten bis ausbleibenden Konsolidierung. Daran ändert auch eine protrahierte Ruhigstellung nichts. Die periostale Überbrückung des Frakturspalts auf der konvexen Seite der Angulation bleibt aus und birgt die Gefahr einer Refraktur in sich [10, 35–37].

Die meisten diaphysären Grünholzfrakturen sind bedingt durch Stürze auf die ausgestreckte Hand mit axialer Krafteinwirkung auf den supinierten Unterarm [34, 36]. Es entsteht eine konvexe Angulation nach palmar, und der Unterarm wird in einer Supinationsfehlstellung gehalten [1, 34]. Die Zugrichtungen des M. pronator teres und des M. supinator neutralisieren sich, und lediglich der M. biceps brachii übt eine flektierende Kraft über den proximalen Radius aus [36]. In einem ambulanten Setting wird nach entsprechender Aufklärung und Analgosedierung unter Bildwandlerkontrolle die Fraktur unter Zug mit einer Pronationsbewegung reponiert, sprich die Handfläche rotiert in Richtung der Fehlstellung (eine adäquate gewichts- und altersadaptierte Schmerztherapie nach institutionellen Richtlinien wird unbedingt empfohlen; [1, 4, 34]). Richtet sich die Konvexität der Angulation nach dorsal, so wird unter Zug supiniert, und die Handfläche rotiert wieder in Richtung der Fehlstellung [1, 4, 34]. Nach erfolgter Reposition muss entschieden werden, ob die Gegenkortikalis durchbrochen wird oder nicht. Dies hängt sicherlich auch von den örtlichen Gegebenheiten des ambulanten Settings sowie der Erfahrung des Ambulanzteams mit dieser Therapieform ab. An der Institution der Autoren stehen 2 Gipszimmer mit Bildwandler und Narkosemaschine sowie einem Gipszimmerarzt und professionellen Gipsassistenten für das Komplettieren einer Grünholzfraktur in Analgosedierung zur Verfügung. Das Überführen einer Grünholzfraktur durch Durchbrechung der Gegenkortikalis in eine potenziell instabile Frakturform birgt immer das Risiko einer sekundären Dislokation in sich. In einem Setting, in dem die Gegenkortikalis im Rahmen der konservativen Therapie in Narkose im OP durchgebrochen wird, stellt sich die Frage, ob gleich eine definitive Versorgung mithilfe der ESIN angestrebt werden sollte. Diese Entscheidungen beschäftigen die Kindertraumatologie schon seit Jahrzehnten.

## Komplettierung der Grünholzfraktur – obligatorisch oder fakultativ?

In ihren Arbeiten hatten Malagaigne schon 1859 und Blount 1942 diese Frage erörtert und die Komplettierung der Grünholzfraktur des diaphysären Unterarms befürwortet. Sie warnten auch explizit vor den Gefahren einer Kompromittierung des interossären Raumes durch verbleibende Achsabweichungen, da diese bleibende funktionelle Einschränkungen zur Folge hätten. Refrakturen innerhalb der ersten 6 Monate nach dem Trauma wurden bei den konservativ behandelten Unterarmschaftfrakturen häufig beobachtet; jedoch wurde kein Zusammenhang mit Grünholzfrakturen als Risikofaktoren hergestellt [3, 38].

Noonan et al. beschrieben zwar den theoretischen Vorteil des Durchbrechens der Gegenkortikalis, da der Kallus vergrößert und dadurch das Risiko einer Refraktur verringert würde, gaben jedoch einer achsengerechten Reposition mit Aufhebung des Drehfehlers den Vorzug [34]. Nach Bould und Bannister haben Frakturen der Unterarmdiaphyse ein 8fach höheres Refrakturrisiko als distale Unterarmfrakturen [39]. Schwarz et al. [40] untersuchten in ihrer retrospektiven Studie die Ursachen für 28 Refrakturen der Unterarmdiaphyse. In ihrer Arbeit zeigten 96 % noch eine Achsabweichung und 84 %, alle initial Grünholzfrakturen, keine vollständige Konsolidierung vor der Refraktur. Die Autoren favorisierten die Komplettierung der Grünholzfrakturen und sahen in der inkompletten Konsolidierung von erfolgten Grünholzfrakturen einen maßgeblichen Risikofaktor für eine Refraktur [40]. Sie traten ähnlich wie Lascombes et al. für eine längere Ruhigstellung bis zu 6 Monate mithilfe von Gips und konsekutiver Schiene ein [40, 41].

Den Stellenwert der inkompletten Konsolidierung als Risikofaktor für eine Refraktur unterstreichen auch die Ergebnisse von Baitner et al. Sie definierten die Refraktur als neuerliche Fraktur an derselben Stelle innerhalb von 18 Monaten. In ihrer retrospektiven Studie wiesen mehr als doppelt so viele Kinder in der Refrakturgruppe Zeichen einer inkompletten Konsolidierung auf als in der Kontrollgruppe (48 % Radius und 67 % Ulna vs. 21 % Radius und 18 % Ulna). Die Häufigkeit der diaphysären Frakturen war in der Refrakturgruppe mit 30 % ebenfalls mehr als doppelt so hoch im Vergleich zur Kontrollgruppe mit 12 %. Die Autoren vermuteten zwar, dass eine persistierende Achsabweichung ein Refrakturrisiko darstellte, konntes dies jedoch nicht signifikant beweisen [42].

Tisosky et al. konnten in ihrer Arbeit eine direkte Beziehung zwischen persistierender Achsabweichung und Zeitpunkt der Refraktur herstellen. Über 60 % der Patient*innen mit einer Fehlstellung > 10° erlitten innerhalb von 6 Wochen eine Refraktur. In ihrem Patientenkollektiv wiesen 31 % eine inkomplette Konsolidierung nach Aufhebung der Ruhigstellung auf. Deshalb propagierten die Autoren eine Gipsruhigstellung für 4 Wochen im Oberarm- sowie 3 bis 4 Wochen im Unterarmgips mit konsekutiver Schienenversorgung bis zur vollständigen Konsolidierung nach durchschnittlich 73 Tagen. Die durchschnittliche Zeit bis zur Refraktur betrug 16 Wochen, und 78 % der Refrakturen betrafen die Diaphyse [43].

In der prospektiven Studie von Schmuck et al. zeigte die Gruppe mit Komplettierung der Grünholzfrakturen zwar eine raschere knöcherne Konsolidierung nach Aufhebung der Ruhigstellung, wies jedoch im Fall einer inkompletten Konsolidierung mit 21 % die höchste Refrakturrate auf. Die Freigabe für alle Aktivitäten erfolgte in 80 % der Fälle aufgrund vermeintlicher radiologischer Konsolidierung, wohingegen die Nachuntersuchung in der Hälfte der Fälle eine inkomplette Ausheilung ergab. Aus der Gruppe der reponierten, aber nichtkomplettierten Frakturen zeigte ein Drittel persistierende Achsabweichungen > 10° im Follow-up. In der Gruppe der komplettierten Frakturen betrug diese Rate nur 7 %, was für ein Durchbrechen der Gegenkortikalis als Prävention gegen Bewegungseinschränkungen spricht. Die Autoren sehen das Durchbrechen der Gegenkortikalis zwar nicht als Risikominimierung einer Refraktur, empfehlen die Komplettierung jedoch bei Überschreitung der Fehlstellung über die altersentsprechende Toleranzgrenzen hinaus. In der radiologischen Kontrolle stellten sich 9 % der Komplettierungen als nicht erfolgreich dar, und nur 5 % wurden nach Durchbrechen der Gegenkortikalis instabil. Die Refrakturen traten durchschnittlich 11 Wochen nach dem Traumaereignis mit einer Häufigkeit von 6,7 % auf [17].

Um die Frage des Einflusses des Komplettierens von diaphysären Grünholzfrakturen des Unterarms auf die Refrakturrate besser beantworten zu können, führten die Autoren des vorliegenden Beitrags nach Genehmigung durch die Ethikkommission der Medizinischen Universität Graz (EK Nummer: 36-332 ex 23/24) in ihrem Patientenkollektiv eine retrospektive Studie aller Kinder und Jugendlichen, die zwischen 2010 und 2023 mit diaphysären Grünholzfrakturen des Unterarms vorstellig wurden, durch. Patientendaten und die Röntgenbilder wurden retrospektiv analysiert, um die Rate des Durchbrechens und die Refrakturrate zu ermitteln.

Für die Datensammlung wurde Microsoft Excel (Microsoft Excel 2021, Microsoft Corporation, Redmond, WA, USA) genutzt. Zur statistischen Auswertung wurde IBM SPS Statistics Version 29.0 (IBM SPS Statistics, IBM Corp., Armonk, NY, USA) eingesetzt. Metrische Daten sind als Mittelwert und Standardabweichung (SD) dargestellt, kategorische Daten als Anzahl und Prozente. Nach Testung auf Normalverteilung mithilfe von Kolmogorow-Smirnow-Tests erfolgte der Gruppenvergleich metrischer Daten durch Anwendung von Mann-Whitney-U-Tests. Gruppenvergleiche kategorischer Daten erfolgten mithilfe des χ^2^-Tests. Die Sankey-Diagramme wurden mit https://sankeymatic.com/ erstellt. *p*-Werte < 0,05 wurden als statistisch signifikant gewertet.

Im Studienzeitraum von 14 Jahren (zwischen 2010 und 2023) wurden 420 Kinder und Jugendliche mit einem durchschnittlichen Alter von 6,9 Jahren (SD ± 3,3 Jahre, Spanne 0 bis 16 Jahre) aufgrund von diaphysären Grünholzfrakturen des Unterarms behandelt. Es waren 61 % (*n* = 255) der Patient*innen männlich und 39 % (*n* = 165) weiblich. Das Alter der männlichen Patienten unterschied sich nicht signifikant vom Alter der weiblichen Patientinnen (m: 7,2 ± 3,2 Jahre vs. w: 6,5 ± 3,2 Jahre, *p* = 0,088, Mann-Whitney-U-Test). Von den Frakturen betrafen 55 % (*n* = 230) die linke Seite und 45 % (*n* = 190) die rechte Seite.

Während bei knapp über 60 % (63,8 %, *n* = 268) der Unterarmfrakturen die Grünholzfrakturen an Radius und Ulna lokalisiert waren, fand sich eine Grünholzfraktur nur des Radius bei 81 (19,3 %) bzw. der Ulna bei 71 (16,9 %) der Patient*innen.

Über die Hälfte der Frakturen (56 %, *n* = 234) wurden konservativ behandelt; bei 44 % (*n* = 186) wurde eine operative Behandlung indiziert. Die operative Therapie bestand bei 98 % (*n* = 183) aus einer Versorgung mithilfe der ESIN. In nur 3 Fällen (2 %) wurde eine Reposition der Fraktur in Narkose mit anschließender Gipsruhigstellung durchgeführt. Im Rahmen der Behandlung wurde bei 223 (53,1 %) der 420 Patient*innen die Grünholzfraktur nicht durchgebrochen; in 197 (46,9 %) Fällen wurde die Therapie mit Durchbrechen der Gegenkortikalis durchgeführt. In diesen 197 Fällen wurde in 105 Fällen (53,3 %) beide Knochen vollständig durchgebrochen, in 52 Fällen (26,4 %) der Radius und in 40 Fällen (20,3 %) die Ulna.

**Abb. 1:**
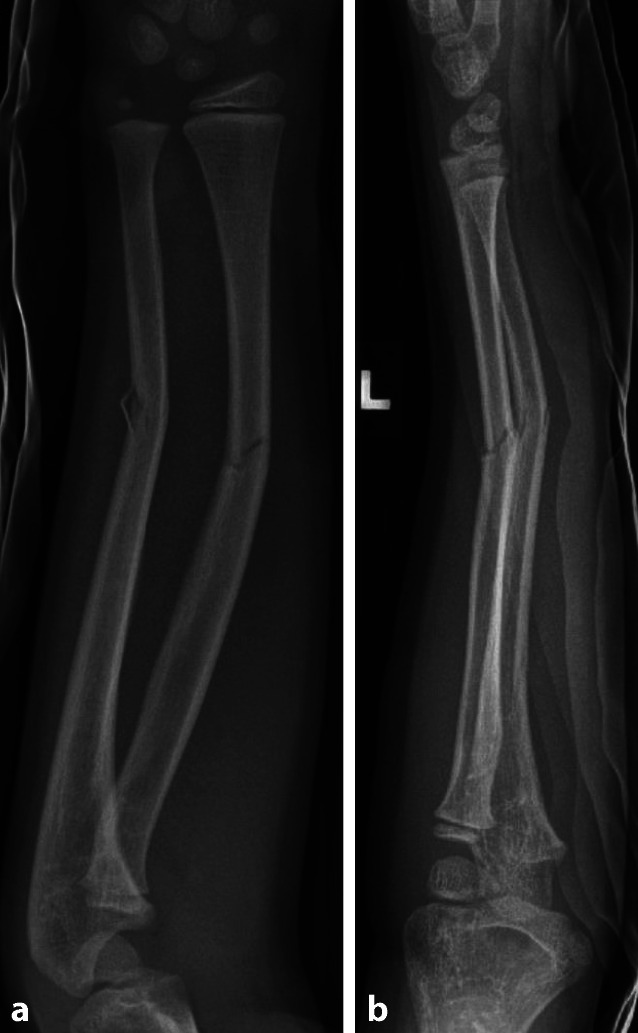
Diaphysäre Grünholzfraktur des Unterarmschafts eines 6‑jährigen Jungen nach Skateboardsturz. **a**, **b** Unfallröntgen in zwei Ebenen

**Abb. 2:**
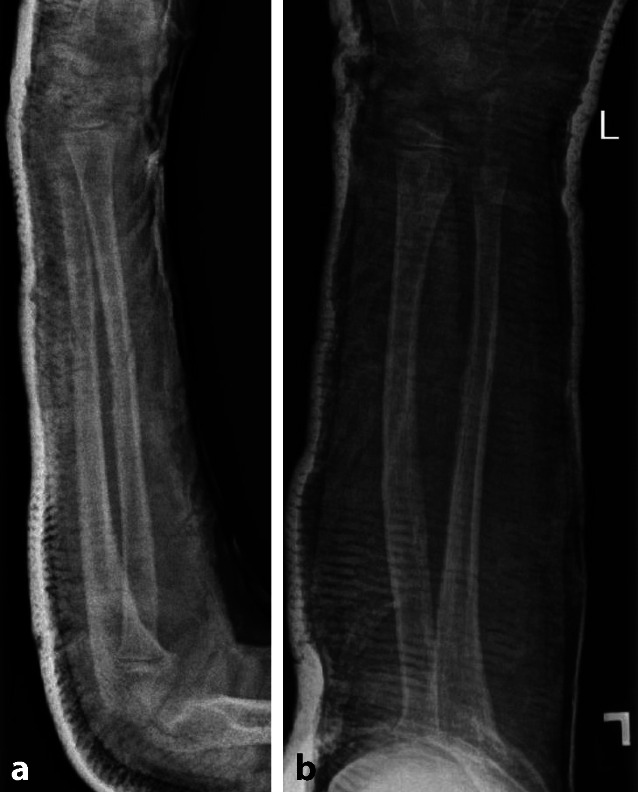
Nach geschlossener Reposition und Gipsruhigstellung; Gegenkortikalis wurde nicht durchgebrochen. **a** seitliche Aufnahme, **b** a.p. Aufnahme

**Abb. 3:**
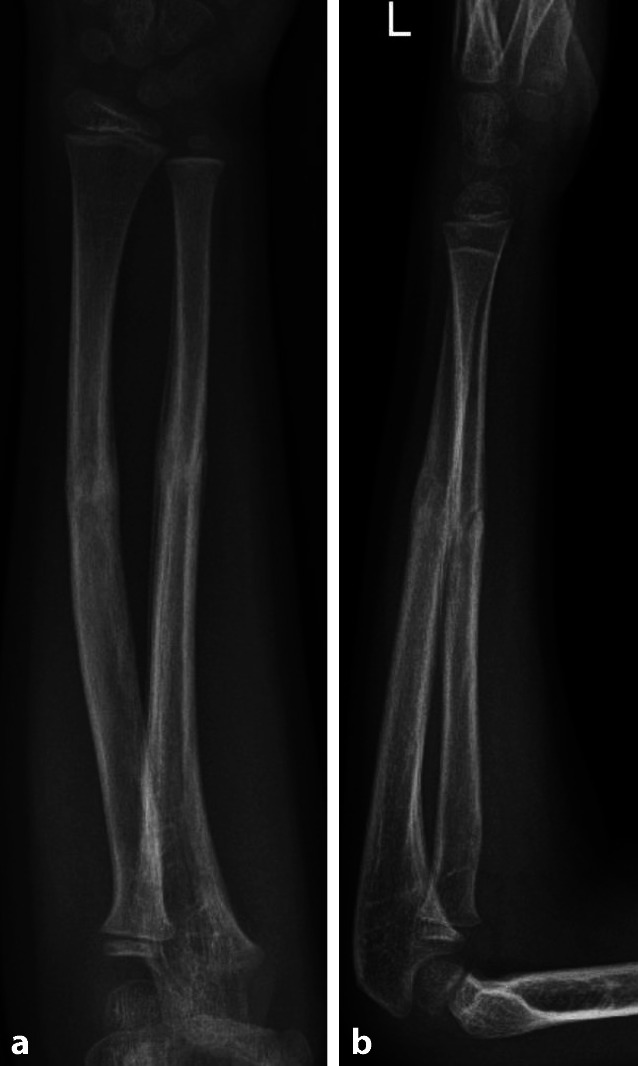
Nach 4 Wochen Gipsruhigstellung gipsfreie Röntgenkontrolle; aufgrund inkompletter Konsolidierung weitere 6 Wochen Sportkarenz. **a** a.p. Aufnahme, **b** seitliche Aufnahme

**Abb. 4:**
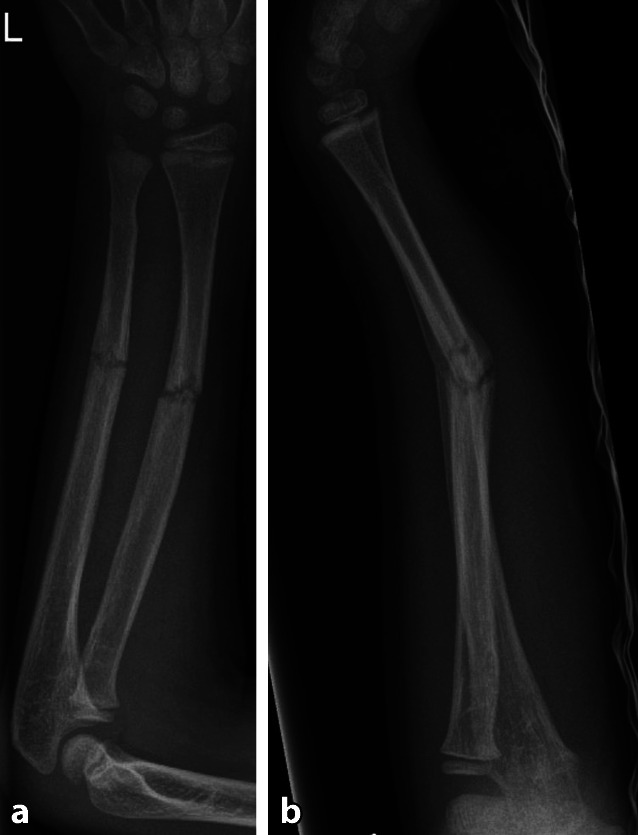
Refraktur 10 Wochen nach initialer Fraktur durch Sturz vom Sofa. **a**, **b** Unfallröntgen in zwei Ebenen

**Abb. 5:**
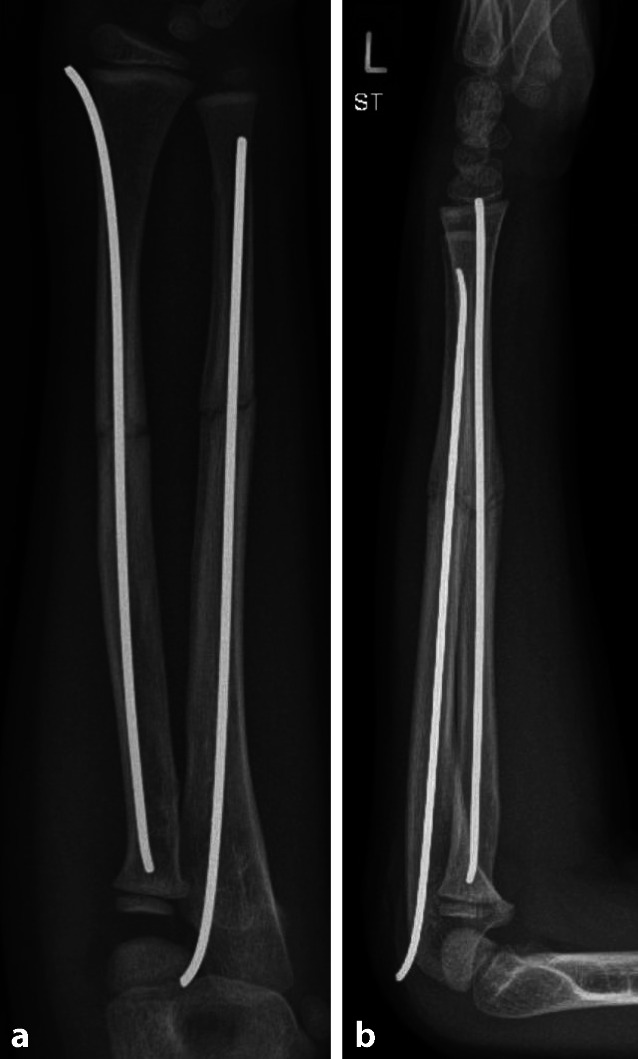
Geschlossene Reposition und Versorgung mithilfe der elastisch-stabilen intramedullären Nagelung (ESIN). **a** a.p. Aufnahme, **b** seitliche Aufnahme

**Abb. 6:**
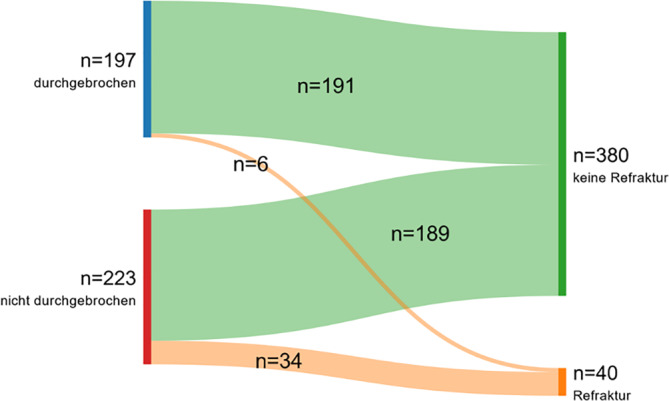
Sankey-Diagramm mit Darstellung der erhöhten Rate von Refrakturen in nichtdurchgebrochenen Grünholzfrakturen

Insgesamt wurden in der Studienpopulation 40 Refrakturen (9,5 %) nach einer durchschnittlichen Zeit von 103 ± 68 Tagen verzeichnet. Das durchschnittliche Alter der Kinder und Jugendlichen mit Refraktur unterschied sich nicht signifikant vom durchschnittlichen Alter der Patient*innen ohne Refraktur (Refraktur 6,3 ± 3 vs. keine Refraktur 6,9 ± 3,4, *p* = 0,232, Mann-Whitney-U-Test). Die Proportion an Refrakturen war signifikant höher in Fällen, in denen die Gegenkortikalis nicht durchgebrochen wurde (nicht durchgebrochen 34 von 223 (15,2 %) vs. durchgebrochen 6 von 197 (3 %), *p* < 0,001, χ2-Test) (siehe Abb. 1, 2, 3, 4, 5). Eine entsprechende grafische Darstellung findet sich im Sankey-Diagramm in Abb. 6.

Der Anteil an Refrakturen war in der operativ behandelten Gruppe signifikant geringer, verglichen mit der konservativ behandelten Gruppe (operativ 6 von 186 (3,2 %) vs. konservativ 34 von 234 (14,5 %), *p* < 0,001, χ2-Test).

**Abb. 7:**
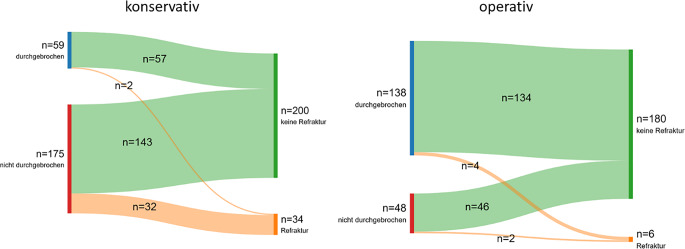
Sankey-Diagramm mit Darstellung der Anzahl der Refrakturen in der konservativ (**a**) und operativ (**b**) behandelten Gruppe in Abhängigkeit vom Durchbrechen der Gegenkortikalis

Um den Einfluss der initialen Behandlung bzw. des Durchbrechens der Gegenkortikalis näher zu beleuchten, wurde eine Subgruppenanalyse durchgeführt. Von den insgesamt 234 konservativ behandelten Frakturen wurden 59 Frakturen in gewichts- und altersadaptierter Analgosedierung durchgebrochen und 175 nicht durchgebrochen. In der Gruppe der komplettierten Frakturen kam es in 3,4 % der Fälle (*n* = 2) zu einer Refraktur und in der Gruppe der nichtkomplettierten Frakturen in 18,3 % (*n* = 32). Dieser Unterscheid war statistisch signifikant (*p* = 0,005, χ^2^-Test). Es wurden 138 der insgesamt 186 operativ behandelten Frakturen durchgebrochen und 48 nicht durchgebrochen. In der Gruppe der durchgebrochenen Gegenkortikalis kam es in 2,9 % der Fälle (*n* = 4) zu einer Refraktur und bei den nichtdurchgebrochenen Frakturen in 4,2 % der Fälle (*n* = 2). Dieser Unterscheid war statistisch nicht signifikant (*p* = 0,668, χ2-Test). Die Abb. 7 stellt diese Unterschiede grafisch dar.

Das Durchbrechen der Gegenkortikalis ist bei konservativer Versorgung zu empfehlen

Der Großteil der Refrakturen (*n* = 33, 82,5 %) wurde mithilfe der ESIN behandelt; nur 7 Fälle (17,5 %) wurden konservativ therapiert. Erneute Probleme bzw. Refrakturen traten in keinem der Fälle auf.

Die Refrakturrate der diaphysären Unterarmfrakturen wird in der Literatur über die Jahre hinweg bis auf wenige Ausnahmen relativ konstant mit 3,2–4,9 % angegeben [39, 43–45]. In der vorgestellten Studie war diese Rate etwas höher (9,5 %), wobei ausschließlich Grünholzfrakturen des diaphysären Unterarms inkludiert wurden. Die höchste Rate der Refrakturen zeigte sich in der konservativ behandelten Patientengruppe, in denen die Gegenkortikalis nicht durchgebrochen wurde. Im Unterschied dazu hatte das Durchbrechen der Gegenkortikalis in der operativ behandelten Patienten keinen statistisch signifikanten Einfluss auf die Refrakturrate. Zusammenfassend kann also festgehalten werden, dass das Durchbrechen der Gegenkortikalis einer Grünholzfraktur des diaphysären Unterarms im Fall eines konservativen Vorgehens empfohlen werden kann, im Rahmen der operativen Versorgung mithilfe der ESIN jedoch keinen Einfluss auf die Refrakturrate zu haben scheint.

## Fazit für die Praxis

- Frakturen des Unterarmschafts sind im Kindes- und Jugendalter häufige Ereignisse; ein Drittel von ihnen sind Grünholzfrakturen.
- Die klassische Grünholzfraktur des diaphysären Unterarms weist immer eine Achsabweichung auf.
- Bei Kindern älter als 10 Jahre dürfen Achsabweichungen > 10° in der Sagittal- und in in der Frontalebene nicht toleriert werden.
- Bei Kinder jünger als 10 Jahre dürfen Achsabweichungen < 15° in der Sagittal- und < 10° in der Frontalebene sowie eine < 50 %ige Translationsfehlstellung toleriert werden.
- Therapieziele sind die Vermeidung der Ausheilung in Fehlstellung und der Erhalt der vollen Funktionalität
- Um die Funktionalität der Umwendbewegungen zu gewährleisten, dürfen proximal der Schaftmitte keine Achsfehlstellungen akzeptiert werden.
- Die inkomplette Konsolidierung der Grünholzfraktur führt zu einer erhöhten Refrakturrate. Die Freigabe für alle Aktivitäten sollte erst nach vollständiger Konsolidierung erfolgen.
- Im Rahmen der konservativen Therapie von Grünholzfrakturen des Unterarmschafts verringert das Durchbrechen der Gegenkortikalis die Refrakturrate.

## References

[1] Caruso G, Caldari E, Sturla FD, Caldaria A, Re DL, Pagetti P et al (2021) Management of pediatric forearm fractures: what is the best therapeutic choice? A narrative review of the literature. Musculoskelet Surg 105(3):225–234 33058085 10.1007/s12306-020-00684-6 PMC8578082

[2] Barvelink B, Ploegmakers JJW, Harsevoort AGJ, Stevens M, Verheyen CC, Hepping AM et al (2020) The evolution of hand function during remodelling in nonreduced angulated paediatric forearm fractures: a prospective cohort study. J Pediatr Orthop B 29(2):172–178 31909747 10.1097/BPB.0000000000000700 PMC7004455

[3] Blount WP, Schaefer AA, Johnson JH (1942) Fractures of the forearm in children. JAMA 120(2):111–116

[4] Jones K, Weiner DS (1999) The management of forearm fractures in children: a plea for conservatism. J Pediatr Orthop 19(6):811–815 10573354

[5] Grahn P, Sinikumpu JJ, Nietosvaara Y, Syvanen J, Salonen A, Ahonen M et al (2021) Casting versus flexible intramedullary nailing in displaced forearm shaft fractures in children aged 7–12 years: a study protocol for a randomised controlled trial. Bmj Open 11(8):e48248 34417215 10.1136/bmjopen-2020-048248 PMC8381323

[6] Lieber J, Schmittenbecher P (2013) Developments in the treatment of pediatric long bone shaft fractures. Eur J Pediatr Surg 23(6):427–433 24327219 10.1055/s-0033-1360460

[7] Mayranpaa MK, Makitie O, Kallio PE (2010) Decreasing incidence and changing pattern of childhood fractures: A population-based study. J Bone Miner Res 25(12):2752–2759 20564246 10.1002/jbmr.155

[8] Sinikumpu JJ, Pokka T, Serlo W (2013) The changing pattern of pediatric both-bone forearm shaft fractures among 86,000 children from 1997 to 2009. Eur J Pediatr Surg 23(4):289–296 23444075 10.1055/s-0032-1333116

[9] Korner D, Gonser CE, Bahrs C, Hemmann P (2020) Change in paediatric upper extremity fracture incidences in German hospitals from 2002 to 2017: an epidemiological study. Arch Orthop Trauma Surg 140(7):887–894 31813018 10.1007/s00402-019-03321-5

[10] von Laer L, Kraus R, Linhart W (2012) Frakturen und Luxationen im Wachstumsalter, 6. Aufl. Thieme

[11] Joeris A, Lutz N, Wicki B, Slongo T, Audige L (2014) An epidemiological evaluation of pediatric long bone fractures—a retrospective cohort study of 2716 patients from two Swiss tertiary pediatric hospitals. BMC Pediatr 14:314 25528249 10.1186/s12887-014-0314-3 PMC4302599

[12] Ryan LM, Teach SJ, Searcy K, Singer SA, Wood R, Wright JL et al (2010) Epidemiology of pediatric forearm fractures in Washington, DC. J Trauma 69(4):S200–S205 20938308 10.1097/TA.0b013e3181f1e837

[13] Naranje SM, Erali RA, Warner WC Jr., Sawyer JR, Kelly DM (2016) Epidemiology of Pediatric Fractures Presenting to Emergency Departments in the United States. J Pediatr Orthop 36(4):e45–e48 26177059 10.1097/BPO.0000000000000595

[14] Lyons RA, Delahunty AM, Kraus D, Heaven M, McCabe M, Allen H et al (1999) Children’s fractures: a population based study. Inj Prev 5(2):129–132 10385833 10.1136/ip.5.2.129 PMC1730475

[15] Rennie L, Court-Brown CM, Mok JY, Beattie TF (2007) The epidemiology of fractures in children. Injury 38(8):913–922 17628559 10.1016/j.injury.2007.01.036

[16] Landin LA (1983) Fracture patterns in children. Analysis of 8,682 fractures with special reference to incidence, etiology and secular changes in a Swedish urban population 1950–1979. Acta Orthop Scand Suppl 202:1–109 6574687

[17] Schmuck T, Altermatt S, Buchler P, Klima-Lange D, Krieg A, Lutz N et al (2010) Greenstick fractures of the middle third of the forearm. A prospective multi-centre study. Eur J Pediatr Surg 20(5):316–320 20577951 10.1055/s-0030-1255038

[18] Joeris A, Lutz N, Blumenthal A, Slongo T, Audige L (2017) The AO Pediatric Comprehensive Classification of Long Bone Fractures (PCCF). Acta Orthop 88(2):123–128 27882802 10.1080/17453674.2016.1258532 PMC5385104

[19] Mehlman CTWE (2015) Diaphyseal radius and ulna fractures: Rockwood and Wilkins’ Fractures in Children

[20] Ploegmakers JJ, Verheyen CC (2006) Acceptance of angulation in the non-operative treatment of paediatric forearm fractures. J Pediatr Orthop B 15(6):428–432 17001251 10.1097/01.bpb.0000210594.81393.fe

[21] Schmittenbecher PP (2005) State-of-the-art treatment of forearm shaft fractures. Injury 36(1):A25–A34 15652933 10.1016/j.injury.2004.12.010

[22] Matthews LS, Kaufer H, Garver DF, Sonstegard DA (1982) The effect on supination-pronation of angular malalignment of fractures of both bones of the forearm. J Bone Joint Surg Am 64(1):14–17 7054197

[23] Tarr RR, Garfinkel AI, Sarmiento A (1984) The effects of angular and rotational deformities of both bones of the forearm. An in vitro study. J Bone Joint Surg Am 66(1):65–70 6690445

[24] Sarmiento A, Ebramzadeh E, Brys D, Tarr R (1992) Angular deformities and forearm function. J Orthop Res 10(1):121–133 1727932 10.1002/jor.1100100115

[25] Colaris J, Reijman M, Allema JH, de Vries M, Biter U, Bloem R et al (2014) Angular malalignment as cause of limitation of forearm rotation: an analysis of prospectively collected data of both-bone forearm fractures in children. Injury 45(6):955–959 24629703 10.1016/j.injury.2014.02.016

[26] Colaris JW, Allema JH, Reijman M, de Vries MR, Biter UL, Bloem RM et al (2014) Which factors affect limitation of pronation/supination after forearm fractures in children? A prospective multicentre study. Injury 45(4):696–700 24182643 10.1016/j.injury.2013.09.041

[27] Weinberg A, Kasten P, Castellani C et al (2001) Which Axial Deviation Results in Limitations of Pro- and Supination Following Diaphyseal Lower Arm Fracture in Childhood? Eur J Trauma 27:309–316

[28] Nakamura T, Yabe Y, Horiuchi Y, Yamazaki N (1999) In vivo motion analysis of forearm rotation utilizing magnetic resonance imaging. Clin Biomech 14(5):315–320 10.1016/s0268-0033(98)90091-2 10521608

[29] Scotcher M, Chong HH, Asif A, Kulkarni K (2023) Radiological Criteria for Acceptable Alignment in Paediatric Mid-Shaft Forearm Fractures: A Systematic Review. Malays Orthop J 17(3):26–32 38107363 10.5704/MOJ.2311.005 PMC10722999

[30] Valone LC, Waites C, Tartarilla AB, Whited A, Sugimoto D, Bae DS et al (2020) Functional Elbow Range of Motion in Children and Adolescents. J Pediatr Orthop 40(6):304–309 32501919 10.1097/BPO.0000000000001467

[31] Fuller DJ, McCullough CJ (1982) Malunited fractures of the forearm in children. J Bone Joint Surg Br 64(3):364–367 7096406 10.1302/0301-620X.64B3.7096406

[32] Daruwalla JS (1979) A study of radioulnar movements following fractures of the forearm in children. Clin Orthop Relat Res 139:114–120 455827

[33] Hughston JC (1962) Fractures of the Forearm in Children. J Bone Joint Surg 44(8):1678–1693

[34] Noonan KJ, Price CT (1998) Forearm and distal radius fractures in children. J Am Acad Orthop Surg 6(3):146–156 9689186 10.5435/00124635-199805000-00002

[35] Gruber R, von Laer L (1979) Zur Atiologie der Refraktur des Vorderarmes im Wachstumalter. Akt Traumatol 9:251–259 44084

[36] Ploss C, Rose S (2016) I. M. Unterarm. In: Marzi I (Hrsg) Kindertraumatologie, 3. Aufl. Springer,

[37] Weinberg AM, Altermatt S, Hell A, Reilmann H (2006) In: Weinberg AM, Tscherne H (Hrsg) Unterarm. Springer,

[38] Malgaigne JF (1859) A Treatise on Fractures, translated from the French by John H. Packard, Lippincott

[39] Bould M, Bannister GC (1999) Refractures of the radius and ulna in children. Injury 30(9):583–586 10707224 10.1016/s0020-1383(99)00151-5

[40] Schwarz N, Pienaar S, Schwarz AF, Jelen M, Styhler W, Mayr J (1996) Refracture of the forearm in children. J Bone Joint Surg Br 78(5):740–744 8836061

[41] Lascombes P et al (1988) Fractures itératives des deux os de l’avant-bras chez l’enfant. Rev Chir Orthop Reparatrice Appar Mot 74 Suppl 2(2):137–139 (Repeat fractures of the 2 forearm bones in children) 3231759

[42] Baitner AC, Perry A, Lalonde FD, Bastrom TP, Pawelek J, Newton PO (2007) The healing forearm fracture: a matched comparison of forearm refractures. J Pediatr Orthop 27(7):743–747 17878777 10.1097/BPO.0b013e318142568c

[43] Tisosky AJ, Werger MM, McPartland TG, Bowe JA (2015) The Factors Influencing the Refracture of Pediatric Forearms. J Pediatr Orthop 35(7):677–681 25436481 10.1097/BPO.0000000000000355

[44] Amilon S, Bergdahl C, Fridh E, Backteman T, Ekelund J, Wennergren D (2023) How common are refractures in childhood? Bone Joint J 105-B(8):928–934 37524339 10.1302/0301-620X.105B8.BJJ-2023-0013.R1

[45] Landin LA (1997) Epidemiology of children’s fractures. J Pediatr Orthop B 6(2):79–83 9165435 10.1097/01202412-199704000-00002

